# An Account of an Operation for Un-United Fracture

**Published:** 1840-03-07

**Authors:** Geo. M‘Cook

**Affiliations:** New Lisbon, Ohio


### AN ACCOUNT OF AN OPERATION FOR UN-UNITED FRACTURE.

BY GEO. M'COOK, M. D.

[Communicated in a letter to Prof. Geo. M'Clellan.]

*Dear Sir*;—I am well persuaded that I cannot render you any better service than to communicate the history of the following operation, which I performed on Mr. John Harman, of Lee township, Carroll county, Ohio. I have permitted twenty months to elapse before communicating the result. I have been awaiting the developments of time, in order that I might be enabled to present the intermediate facts, so as to enable you to judge of the merit or demerit of the operation.

Mr. Harman is a blacksmith, thirty-six years old, of temperate habits, possessing a good constitution, without any trace of hereditary predisposition to disease. He got his left thigh bone broken by the fall of a limb of a tree, in April, 1836. The limb was fractured obliquely, about the lower part of the upper third of the bone. I forbear to detail the manner in which the limb was reduced and dressed. I will observe, however, that Bowen's splint (constructed chiefly on the principle of Amesbury's) was applied, and that either its action was misconceived, or that some officious attendant must have interfered with its original application. I was called, in conjunction with six highly respectable professional gentlemen, to see the limb, in September, about five months after the occurrence. Bowen's splint, as we learned, had been continued all this time. There was free motion in that part of the limb corresponding to the fracture; pressure over the end of the bone gave no pain; motion in either direction gave no pain. The ends of the bone presented indubitable evidences of enlargement. The knee was anchylosed. A comparison of the broken limb with the length of the sound one, proved the fact, that the ends of the fractured bone were three inches apart. We were all united in the opinion, that there was no beneficial prospect in the continuance of any apparatus to the limb, as it then was. We were all of opinion that some operation must be performed, in order to bring about a reunion. The means, however, which should be resorted to, to effect this purpose, was the grave question to decide. Two operations, and but two, presented themselves to our consideration. The first was that communicated to the surgical world, by the father of American surgery, for false joint. The second was that of Mr. White of Manchester. In my preference for the latter operation, I stood alone. *I was overruled.* If I had recognised the case as *a false joint*, the authority cited and the precedent, would have been as decisive in my mind as it was in that of the other gentlemen. Was this a false joint? I think not. This consists in the fractured ends of the bone *being united* by *an intervening substance*, differing in *texture* and *flexibility* from bone. The ends of this bone had formed separate attachments; there was no juncture. I did not consider that *one requisite* of a joint, *motion*, was sufficient to constitute it such in my sight. The relation in which the fractured extremities stood to each other—the one on the anterior part of the thigh, the other being drawn upward and outward—forbade the idea of hopeful success, by the intervention of the seton. The excessive irritation which must have attended the absorption of the ossific matter, deposited on the ends of the bone, and the debility consequent to a protracted discharge, were prospective objections in my mind. If these arguments were tenable in a sound constitution, they would be more so when considered in reference to a constitution already shaken by the force of disease. I do not wish to be considered as arrogating to myself any superiority over the other medical gentlemen, from whom I unhappily differed. No such thing. They were considerate, they were honest, they merit a high standing in their profession, and the difference between us in this case was no greater than might occur between honest men, in matters of ambiguity. And I will take the liberty of saying, that their reverence for the departed father of surgery had a strong bias, and justly too, on their minds, and evinces the correctness of the motto "*Non jurare in verbum majistri.*" But to return. At the unanimous request of all present, I introduced the seton on the 11th of September, 1836. The limb, with a view to steadiness alone, was replaced in Bowen's splint. The patient was ordered a thorough stimulating and tonic course, consisting of bark, quinine, wine of opium, carb. ammon, etc.; a full diet. The seton was continued until 20th of December, at which time I was called on to visit Mr. Harman. I found him labouring under great constitutional irritation; he had night sweats, colliquative diarrhœa, the usual concomitants of hectic. The matter was profuse and illy digested. I now acted on my own responsibility. I did not hesitate to withdraw the seton, and prescribe a renewed, strongly stimulating and tonic course.

He was kept faithfully under this treatment until Monday, the 19th day of May, 1837. He came to my house on Saturday the 17th. On the 18th, his bowels not being very free, he took a *laxative*. He had now come to submit to Mr. White's operation, or *any other* which I might think fit to adopt. The preceding night he took half a grain of the sulph. morph. You will not think it strange that I was surprised at seeing him still willing to undergo a severe operation, and that, too, under circumstances by no means flattering in their promise. I was not so sanguine, at this moment, of the efficacy of the operation proposed. I viewed, with solemn consideration, the responsibility which was about to attach to its performance. Although I had, on two different occasions, performed the operation successfully, on the lower limb, I felt disposed to shrink from it in this case. I disclosed to my patient and his wife the difficulty and the extreme danger of the operation. At every step we advanced in conversation, instead of creating doubt and hesitancy in his mind, I found an increased degree of confidence and determination. I had invited Drs. Vallendigham of this village, and Lewis of Carroll county, to witness the operation. I was kindly assisted on this occasion by both. We placed our patient on a narrow, firm bed, suitably prepared. On Monday morning I commenced my operation by making an incision five inches long, parallel with the fibres of the rectus, cutting down boldly to the bone. I then introduced a scalpel, guarded by my right index finger, until I came in contact with the end of the bone, which had formed fine fibro-cartilaginous attachments to the muscles. I followed up the attachments with the knife, until I broke them up, and gave mobility to the bone. I then ordered the leg to be moved inward, and so became enabled to insert underneath the bone, a narrow piece of smoothly planed pine shingle, by which I protected the soft parts, and gave steadiness to the bone. With the common amputating saw, I took off nearly one inch of the femur. My assistants then turned the patient on his side, but found it necessary to remove the tourniquet, which I had applied to restrain hæmorrhage, in case I should accidently cut a considerable branch of the profunda; without so doing, I could not have carried my incision as far upward as was desirable. I then made a deep and long incision on the under and outer side of the femur, introduced my scalpel as before, broke up all the adhesions, and caused the limb to be carried inward, to evert the femur, of which I took off one inch and a half by the trephine and bone nippers, the only instruments which I found available in this stage of the operation. After waiting a considerable time to stimulate, and to permit the small vessels to contract, I reduced the bone, and adjusted the ends as nearly as possible—washed the wounds, and applied three ligatures and a requisite number of adhesive strips to each wound. After applying the many-tailed bandage, and long cushions, I placed the limb in Wood's animo-metallic splint. I preferred this to Physick's modification of Desault, for the reason that I did not expect to require strong extension and counter-extension; and I looked upon the flexure of the pelvis on the thigh as essential to counteract the action of the psoas magnus and iliacus internus. In accomplishing this object, I met with considerable difficulty. The wounds healed kindly in two weeks; the discharge was healthy in quantity and in quality. My patient was kept under a thorough tonic and antispasmodic treatment all the time. The limb evinced evident signs of union in five weeks; in eight weeks it could be moved with safety; in three months he returned home, with injunction to permit six months to elapse before he would attempt to use the limb. On two different occasions exfoliation and discharge of small spicula of bone took place.

I saw Mr. Harman, a few weeks ago, and he walks very well with the use of a common cane. He informed me, that on the day before our interview, he shod five horses. He enjoys good health. The limb is two inches and a half shorter than the sound one. About twenty five minutes were occupied in the cutting and amputating processes of the operation. Two hours elapsed, however, or thereabouts, from the commencement, until he was dressed and put in bed. If I had occupied less time, and had not fully administered my stimulants, my patient must have incurred more risk, or perhaps have died in the operation. *The pain was indescribable*—books may give rules for the performance of such operations, and may give faint ideas of the suffering to be endured, but let me assure you, they fall far short of conveying the idea of their horrors. If this operation had been performed by yourself or some other experienced surgeon, at home, where you are surrounded by all that is desirable under similar circumstances, its frightful character might bear a different aspect. I would not wish to arrogate any very superior qualification for such emergencies, but I may say, without the charge of egotism, any less nerve, than I possess, would have rendered me unfit for this operation. The amount of blood lost, did not exceed half a pint. My patient under a full dose of morphia rested well the after part of the day and succeeding night. No circumstance of an unpleasant character occurred during convalescence, except an attack of cholera morbus, induced by an overcharge of the stomach. From my entrance on my profession, I have been taught to place a a great estimate on a good constitution and a sound moral condition; my present operation has taught me a fuller lesson on the subject. I am well persuaded that if Mr. Harman had possessed less calmness, less patience, and less determination, he must have fallen a victim to the operation. Your liberality, sir, will excuse the defects of this communication; like too many of my professional brethren, I write but little; and must of necessity fail in those traits which pertain to this kind of experience. When you have examined this case you will be pleased to hand it to Dr. Biddle for his special supervision. I have full confidence that he will do it justice, and if there is any thing in it worthy of publicity, it is at his service. Before I conclude I will only say, that if I amputate the leg, (measuring thirty inches above the knee,) spoken of in my late letter to Dr. Hamilton, I will preserve it, and send it to you. I take it to be osteosarcoma.

*New Lisbon, Ohio, Jan.* 28, 1840.

